# ENGAGEMENT OR ENJOYMENT? ATTITUDES OF OLDER ADULTS IN GROUP LUNCH VERSUS. LIFELONG LEARNING PROGRAMS

**DOI:** 10.1093/geroni/igad104.1181

**Published:** 2023-12-21

**Authors:** Su-I Hou

**Affiliations:** School of Global Health Management & Informatics, University of Central Florida, Orlando, FL, USA, Oviedo, Florida, United States

## Abstract

**Purpose:**

This study examines aging attitudes among older adults who participated in the neighborhood lunch (NLP) and lifelong learning (LLP) programs.

**Methods:**

Older adults from an NLP and an LLP in the same county in Florida participated. An aging attitudes measurement originally developed by German researchers was used.

**Results:**

A total of 193 older adults participated, 43% from NLP. The mean age was 73.2 (SD=7.78) years. Data showed that LLP older adults endorsed higher on “would like to have responsibilities,” “have a task,” “do unpaid volunteer work,” and “help others” (p<.001). NLP participants, on the other hand, endorsed higher on “no longer have to contribute to society,” “want to enjoy life,” and “finally want to rest and relax” (all p<.001). Exploratory factor analyses (EFAs) using principal component extraction methods and varimax rotation with Kaiser normalization showed a clear balanced 2-factor pattern, with total variance explained 60% (30% each). These two factors were consistent with the theory representing engagement versus enjoyment aging attitudes. Reliabilities of the two-factor scales were satisfactory, with Cronbach’s alpha of .764 (CITC ranged: .457 ~ .660) for the engagement scale (4-item) and.634 (CITC ranged: .379~.499) for the enjoyment scale (3-item).

**Discussion:**

Current findings showed that LLP participants endorsed higher engagement, while NLP participants endorsed higher enjoyment towards aging. This tested aging attitudes scale consists of 7 items with two clear theory-consistent factors among community-dwelling older adults. Results can help researchers quickly assess aging attitudes of diverse groups of older adults for tailored program and service development.

